# Quantification of pulmonary edema in heart failure using MRI: invasive validation and evaluation in HFpEF and HFrEF patients

**DOI:** 10.1186/1532-429X-18-S1-O49

**Published:** 2016-01-27

**Authors:** Richard B Thompson, Kelvin Chow, Viktor Sekowski, Evangelos Michelakis, Joseph J Pagano, Wayne Tymchak, Mark Haykowsky, Justin Ezekowitz, Gavin Y Oudit, Jason Dyck, Ian Paterson

**Affiliations:** 1 grid.17089.37Biomedical Engineering, University of Alberta, Edmonton, AB Canada; 2 grid.17089.37Medicine, University of Alberta, Edmonton, AB Canada; 3 grid.17089.37Pediatrics, University of Alberta, Edmonton, AB Canada; 4 grid.267315.40000000121819515College of Nursing and Health Innovation, University of Texas at Arlington, Arlington, TX USA

## Background

Pulmonary edema is a cardinal feature of heart failure (HF), reflecting impaired ventricular filling. The associated increase in left ventricular end-diastolic pressure (LVEDP) results in accumulation of fluid in the interstitium/alveolae. MRI signal is directly proportional to water density (WD) and is thus an attractive tool for quantitative assessment of edema. The primary goals of the current study were to: (1) evaluate the relationship between MRI-derived lung WD and invasively measured LVEDP in patients with HF and (2) characterize lung WD in healthy controls, patients at risk for HF and HF patients with NYHA class I-III symptoms.

## Methods

Consecutive patients with HF referred for a diagnostic cardiac catheterization (LVEDP or wedge pressure measured) were screened for enrollment in the validation arm of the study (19 patients recruited). Patients underwent MRI within 2 hrs of catheterization for comparison of MRI-derived lung WD and filling pressures. 226 additional subjects from the Alberta HEART study (BMC Cardiovasc Disord. 2014 Jul 25;14:91) included: healthy controls N = 56, at-risk for HF N = 58, HF with preserved LVEF (HFpEF) N = 64 and reduced LVEF (HFrEF) (< 50%) N = 48, who were evaluated with the same lung water imaging protocol.

Imaging was performed on a Siemens Sonata 1.5T (Siemens Healthcare, Erlangen, Germany). Lung water was measured using a half-Fourier single-shot turbo spin echo (HASTE) pulse sequence. 128 × 66 matrix, 8 mm slice, 4/8 ths partial Fourier, 780 Hz/pixel bandwidth, 12 ms TE, image acquisition during diastasis, body coil excite/receive. Lung image signal intensities from a single sagittal slice in the right lung were normalized to units of water density using a liver region as a reference signal (70% liver WD assumed, *J Appl Physiol*. 1959;14:1005-8).

## Results

**Figure 1:**
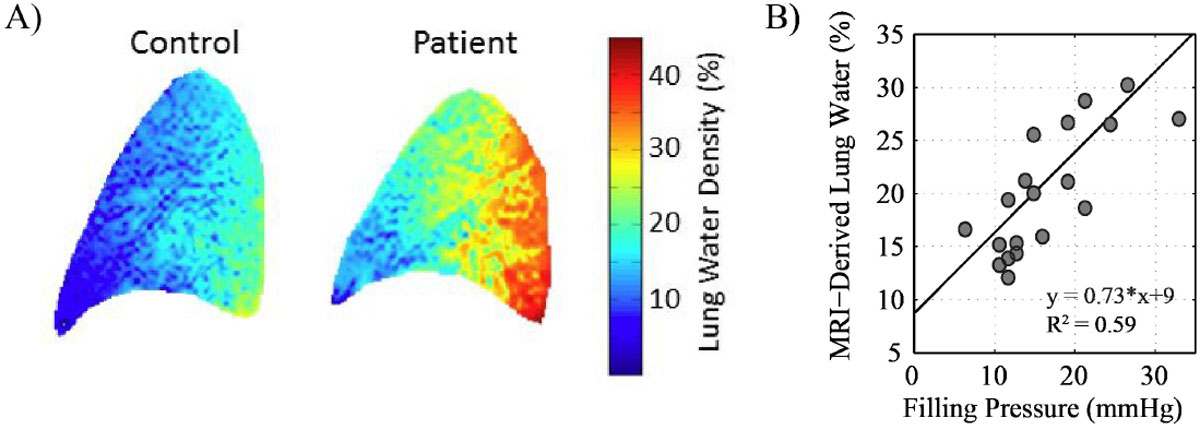
**Sample lung water images in a healthy control (left, WD = 14.0%) and HFrEF patient (right, WD = 24.5%, LVEDP = 31 mmHg)**. B) Correlation of MRI-derived filling pressures and invasively measured filling pressures in HF patients (p < 0.01).

**Figure 2:**
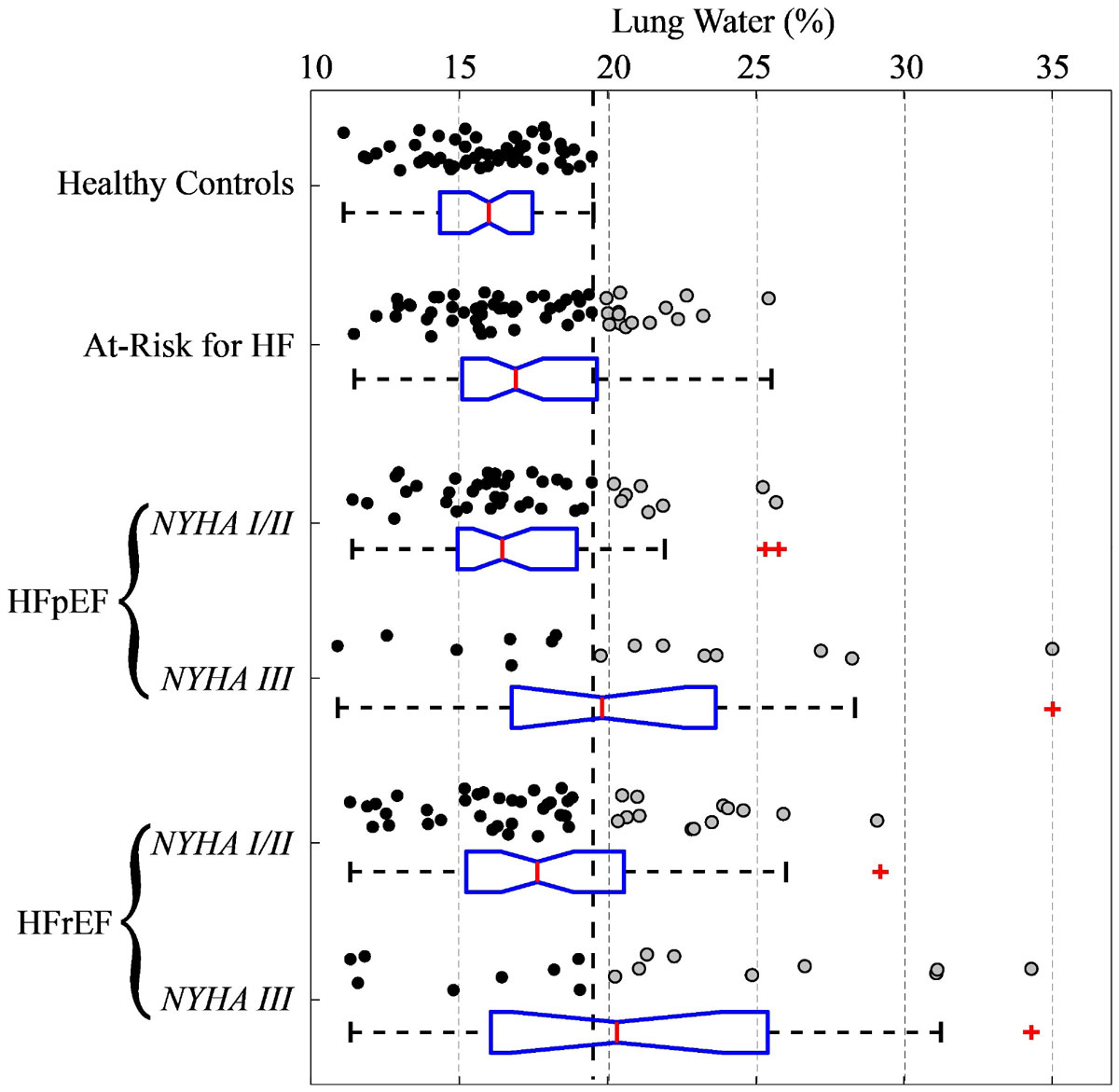
**Summary of lung water content (WD) in all subjects from the Alberta HEART cohort (HFpEF is heart failure with preserved ejection fraction and HFrEF is heart failure with reduced ejection fraction)**. Each circle is an individual subject (gray for lung water > 19.5% which is the maximum healthy control value - vertical dashed line), and a box-plot for each group shows the median, 25% and 75% and full extent of the data, with outliers shown as red crosses. NYHA III groups have significantly increased lung WD as compared to healthy controls (P < 0.05). All groups were similar for age and gender.

Fig. 1A shows sample lung water density images from a representative control and a HF subject. Fig. 1B shows the significnat correlation between lung water and filling pressures in the validation cohort (p < 0.01). Fig. 2 shows lung WD for all subjects in the Alberta HEART cohort as well as box plots for each group. Those with NYHA Class III have significantly increased lung WD as compared to controls (P < 0.05). Individuals with increased lung water (WD that exceeded all control subjects, beyond dashed line) are shown in gray.

## Conclusions

Increased lung WD is significantly associated with increased filling pressures in HF patients, and WD can be estimated as part of a standard clinical CMR exam. Increased lung water is associated with worsening NYHA Class, independent of LVEF.

